# PM-CNN: microbiome status recognition and disease detection model based on phylogeny and multi-path neural network

**DOI:** 10.1093/bioadv/vbae013

**Published:** 2024-01-27

**Authors:** Qiangqiang Wang, Xiaoqian Fan, Shunyao Wu, Xiaoquan Su

**Affiliations:** College of Computer Science and Technology, Qingdao University, Qingdao 266071, China; Department of Gastroenterology, Shouguang Hospital of Traditional Chinese Medicine, Weifang 262700, China; College of Computer Science and Technology, Qingdao University, Qingdao 266071, China; College of Computer Science and Technology, Qingdao University, Qingdao 266071, China

## Abstract

**Motivation:**

The human microbiome, found throughout various body parts, plays a crucial role in health dynamics and disease development. Recent research has highlighted microbiome disparities between patients with different diseases and healthy individuals, suggesting the microbiome’s potential in recognizing health states. Traditionally, microbiome-based status classification relies on pre-trained machine learning (ML) models. However, most ML methods overlook microbial relationships, limiting model performance.

**Results:**

To address this gap, we propose PM-CNN (Phylogenetic Multi-path Convolutional Neural Network), a novel phylogeny-based neural network model for multi-status classification and disease detection using microbiome data. PM-CNN organizes microbes based on their phylogenetic relationships and extracts features using a multi-path convolutional neural network. An ensemble learning method then fuses these features to make accurate classification decisions. We applied PM-CNN to human microbiome data for status and disease detection, demonstrating its significant superiority over existing ML models. These results provide a robust foundation for microbiome-based state recognition and disease prediction in future research and applications.

**Availability and implementation:**

PM-CNN software is available at https://github.com/qdu-bioinfo/PM_CNN.

## 1 Introduction

Microbial communities inhabit various human body parts, including gut, oral cavity, skin, reproductive tract (Turnbaugh *et al.* 2007, Huttenhower *et al.* 2012, Yatsunenko *et al.* 2012), etc. These microorganisms coexist with the human body, exerting a profound influence on human health and disease development (Clemente *et al.* 2012, Blaser 2014, Julian *et al.* 2016). Since the inception of the Human Microbiome Project (Peterson *et al.* 2009), there has been a pervasive fascination with unraveling the intricate interactions between the intestinal and oral microbiomes and their human host (Ngom-Bru and Barretto 2012, Herremans *et al.* 2022). Numerous studies have demonstrated strong associations between certain diseases and the human microbiome (Zhang and Zhang 2013, Belkaid and Hand 2014, Qin *et al.* 2014). This is particularly evident in the pathogenesis of chronic conditions such as inflammatory bowel disease, intestinal diarrhea, gingivitis, and periodontitis (Kostic *et al.* 2014, Guarino *et al.* 2015, Cheng *et al.* 2017). For example, individuals with inflammatory bowel disease (IBD) demonstrate a declining trend in gut microbiome biodiversity compared to their healthy counterparts (Halfvarson *et al.* 2017). These patterns within microbiomes hold the potential to differentiate between healthy and diseased states, offering prospects for precision medicine in numerous microbiome-related conditions.

Microbiome feature data are typically characterized by high-dimensionality, over-dispersion, and sparsity (Lozupone *et al.* 2012).

Consequently, machine learning (ML) techniques have been widely employed to uncover the connections between microbial dynamics and human health status (Forslund *et al.* 2015, Pasolli *et al.* 2016, Qu *et al.* 2019, Fenglong *et al.* 2021). These techniques include random forest (RF) (Tin Kam 1995), support vector machine (SVM) (Suthaharan 2016), k-nearest neighbors (KNN) (Cover and Hart 1967), extreme gradient boosting (XGBoost) (Chen *et al.* 2016), decision trees (DT) (Quinlan 1986), among others. Given that microbes exist in symbiotic communities with frequent interactions, considering phylogeny, which quantifies the evolutionary or functional relations among microbes, is crucial when processing microbiome data. However, most ML models overlook these inter-microbial relationships, limiting their efficacy in status classification.

Recently, deep learning (DL) and neural network (NN)-based approaches have been successfully adapted from image analysis and natural language processing to microbiome research to address the challenges of classifying the status of complex microbial structures. One such method, ph-CNN (Fioravanti *et al.* 2017), utilizes a convolutional NN-based deep learning approach for binary classification, focusing on the neighborhood of microbes within a phylogenetic tree. While this approach shows promise, there is mounting evidence suggesting that many diseases share overlapping microbiome signatures (Gacesa *et al.* 2022). Consequently, binary classifiers can produce erroneous results when applied to real datasets confounded by unrelated diseases (Su *et al.* 2022). Moreover, the computational complexity involved in searching for microbial neighbors limits its scalability for high-dimensional microbiome data. MDeep (Wang *et al.* 2021) improved upon ph-CNN by introducing a dimension reduction prediction method, resulting in greater efficiency and reduced runtime. However, this method simulates convolutional layers based on the taxonomy hierarchy levels, potentially missing quantitative genotype relations across microbes, and it remains limited to binary classification.

**Figure 1.**
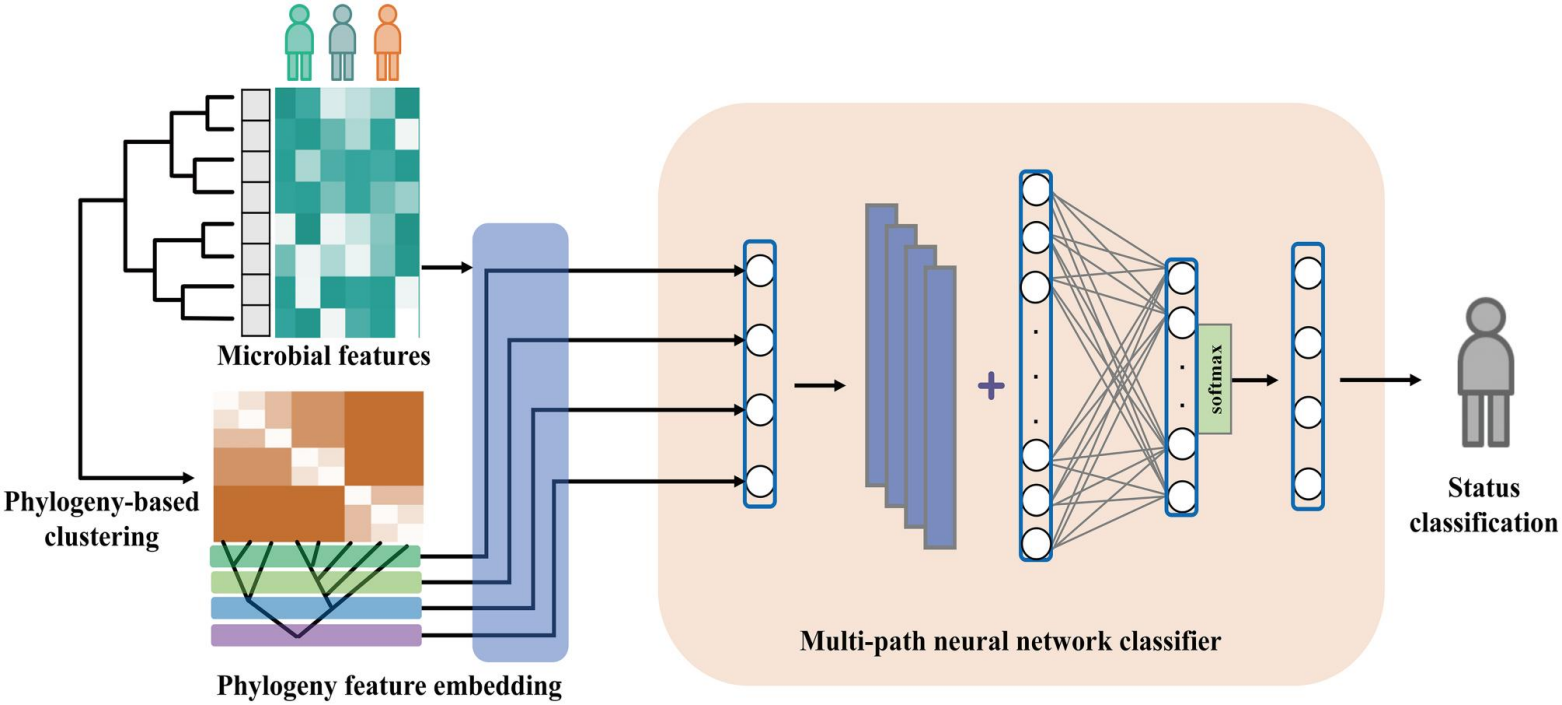
Workflow of the PM-CNN. The microbial richness features are embedded with phylogeny by multi-layer hierarchical clustering, then a multi-path neural network learns such information to make detection for status detection and classification.

In this work, we propose a phylogeny-based neural network model called PM-CNN (Phylogenetic Multi-path Convolutional Neural Network) for the classification of multiple statuses and the detection of diseases using microbiome data (Fig. 1). Leveraging a phylogenetic structure, PM-CNN re-organizes all microbes into different groups and then constructs a multi-path convolutional neural network to quantify the relationships among microbes. Subsequently, an ensemble learning method fuses the features learned by the multi-path CNN (Convolutional Neural Network) to make classification decisions. The application of PM-CNN to human gut and oral microbiome datasets demonstrates its superior performance in multi-status classification compared to other existing ML models. This performance provides a robust foundation for microbiome-based state recognition and disease prediction in future studies.

## 2 Algorithm design

### 2.1 Quantitative relations among microbes induced from phylogeny

**Figure 2.**
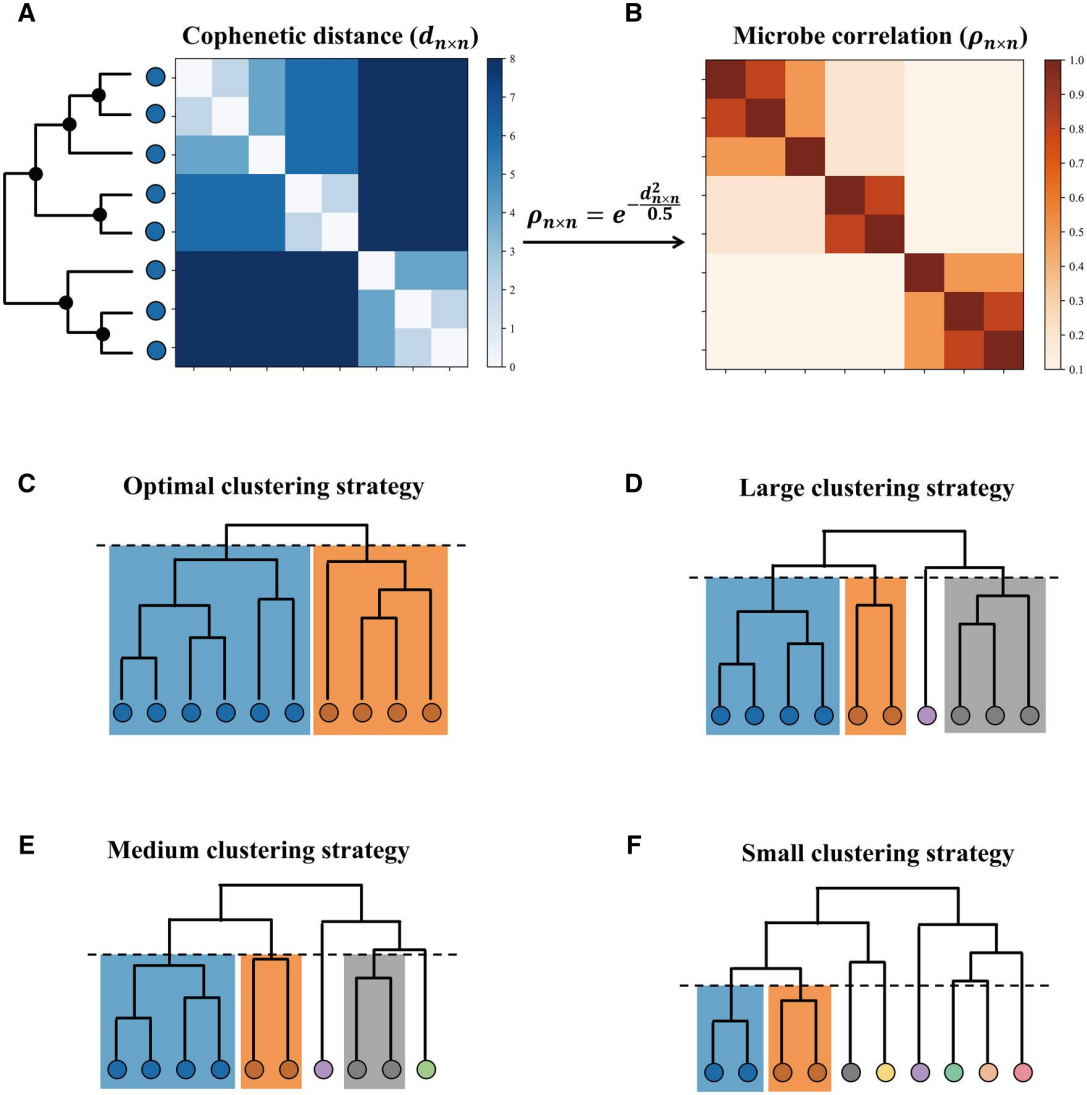
Multi-layer hierarchical clustering of microbes based on phylogeny. (A) The pairwise cophenetic distances among microbes are parsed from their common phylogenetic tree. (B) Normalized quantitative relations among microbiome are transformed from the distance matrix by a Gaussian kernel function. (C–F) Hierarchical clustering is performed based on the normalized quantitate relation with optimal strategy (C), large clustering strategies (D), medium clustering strategy (E) and small clustering strategy (F).

After microbiome profiling (refer to the Section 5 for details), microbiome samples were represented using microbial features such as operational taxonomy units (OTU), amplicon sequence variants (ASV), and taxonomic information derived from sequences. Microbial features form a relative abundance matrix with dimensions of *n *×* m*, where *n* represented the number of samples and *m* was the number of microbes. To assess the phylogenetic relationships between pairs of microbes, we calculated the distance between each pair using the cophenetic metrics (Felsenstein 1985), which measured the branch lengths to their common ancestor in the phylogenetic tree (Fig. 2A; refer to the Section 5 for details). This process was repeated for all possible pairs of microbes, resulting in the generation of a pairwise cophenetic distance matrix *d_n×n_*. Finally, the quantitative relation of all microbes $\rho_{n\times n}$ can be normalized from the cophenetic matrix by the Gaussian kernel transformation (Fig. 2B), which was defined as Equation (1):

(1) $$\rho_{n\times n}=e^{-\frac{d_{n\times n}^{2}}{0.5}}.$$

### 2.2 Multi-layer hierarchical clustering based on pairwise relations

Based on the normalized relation matrix $\rho_{n\times n}$, microbes with high correlation were grouped by the hierarchical clustering algorithm. During the computing procedure, we set the clustering layer with highest silhouette coefficient (Rousseeuw 1987, Tibshirani *et al.* 2001) of the number of groups as the optimal clustering result (Fig. 2C). In addition, to make full use of the phylogenetic structure among microbes for the status classifier, we also kept clustering results under multiple layers (Fig. 2D–F). Specifically, we defined the large, medium and small clustering strategies, in which each cluster contained 1/20, 1/100, and 1/500 of the total number of all microbial features on average, respectively. According to the expected number of different clustering strategies, we set the corresponding parameter size in the hierarchical clustering algorithm. Thus, based on the four clustering results (i.e. optimal, large, medium, and small), the microbial relative abundance table can be sorted into four different forms with phylogeny information.

### 2.3 Multi-status classification model based on multi-path neural network

**Figure 3.**
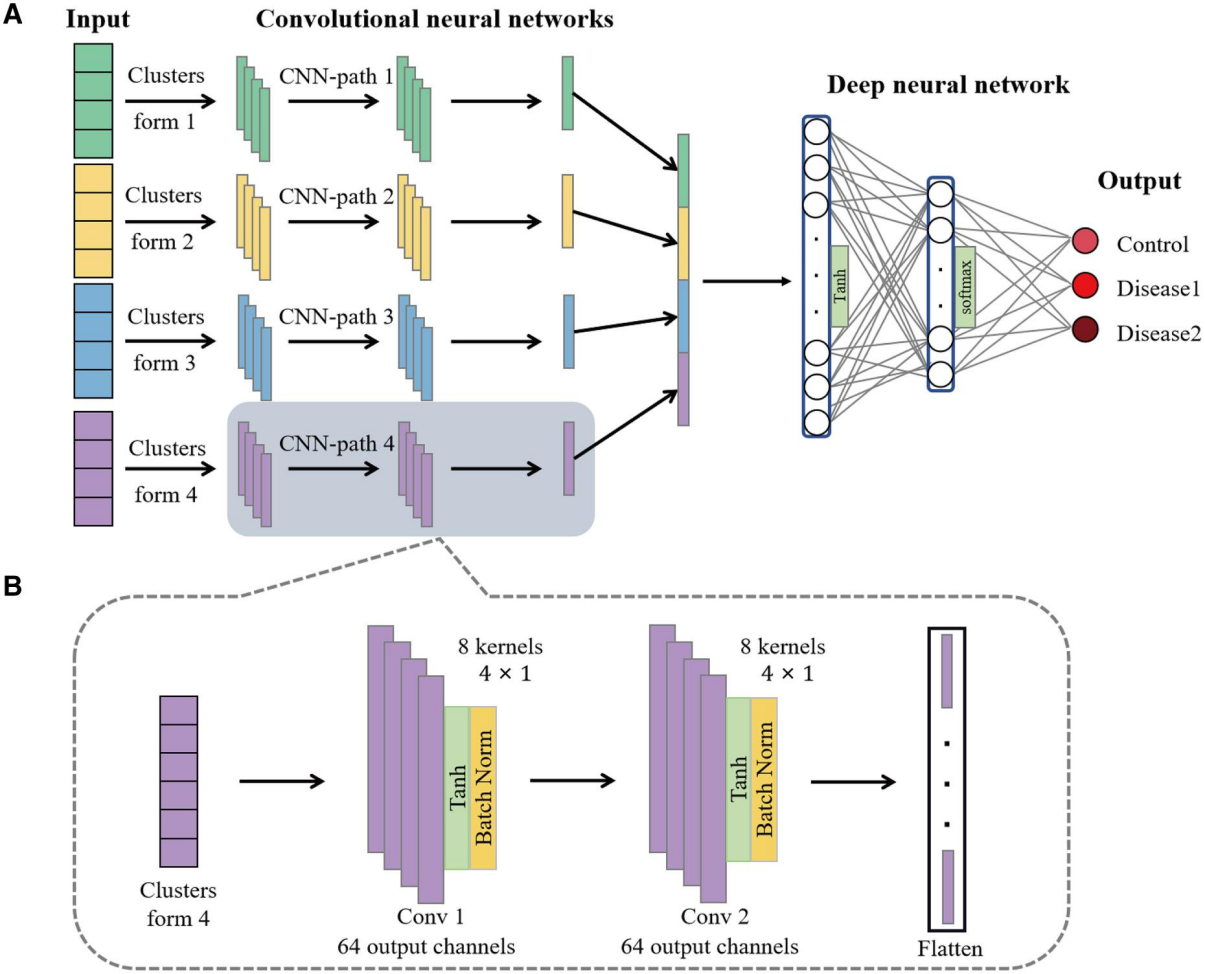
Multi-status classification model based on multi-path neural network. (A) Multi-path neural network model in the PM-CNN framework. (B) A single-path convolutional neural network.

Using the input training data consisting of microbial relative abundance table in four clustering forms and a metadata file of status, PM-CNN invoked an ensemble-learning-based model for multi-status classification (Fig. 3A). This model consisted of a four-path convolutional neural network and a deep neural network (i.e. two fully connected layer). Each path of CNN extract features from the input microbial table in a specific form. Specifically, to effectively capture the phylogenetic correlation between microbial species, in each path we used 16 1D convolution filters with a stride size of 4 and an input and output channel number of 64 (Fig. 3B). Thus, the multi-path CNN not only comprehensively captured the microbiome features with phylogenetic relatedness, but also alleviated the inherent high-dimensional challenges of microbiome data, which potentially enhanced the classification performance. In addition, we also set a batch normalization (Ioffe and Szegedy 2015) and a Tanh activation function (Mcculloch and Pitts 1943) in the model to improve the convergence speed and nonlinearity of the model. After that, features obtained from each path CNN were flattened into a single vector, then interpreted and sent to the deep neural network to make classification decision. The fully layer layers were also enhanced by the Tanh activation function and normalization. Finally, PM-CNN produced the classification results for each category based on their probability distributions obtained from the softmax function (Hinton and Salakhutdinov 2006).

## 3 Results

### 3.1 Experiment design

To assess the performance of our PM-CNN model in status classification and disease detection, we utilized two real microbiome datasets (Table 1) for benchmarking. Dataset 1 comprised a total of 1587 human oral 16S amplicon microbiomes, distributed across 653 healthy controls, 660 gingivitis samples, and 274 periodontitis samples. Meanwhile, Dataset 2 consists of 3113 human gut 16S amplicon microbiomes, encompassing 1418 healthy controls, 993 samples from individuals with chronic gut inflammation (IBD), 360 samples from human immunodeficiency virus (HIV) patients, 222 samples from individuals with gut diarrhea disease (EDD), and 120 samples from patients with colorectal cancer (CRC). All microbiome sequences underwent preprocessing using the Parallel-Meta Suite software (Chen *et al.* 2022) (please refer to the Section 5 for a detailed description of this process).

**Table 1.** Information of microbiome datasets.

Status	Number of samples
(A) Dataset 1 (human oral microbiome)	
Periodontitis (Griffen *et al.* 2012, Abusleme *et al.* 2013, Kirst *et al.* 2015, Corrêa *et al.* 2017)	274
Gingivitis (Huang *et al.* 2014)	660
Healthy control (Peterson *et al.* 2009, Griffen *et al.* 2012, Abusleme *et al.* 2013, Huang *et al.* 2014, Kirst *et al.* 2015, Corrêa *et al.* 2017)	653
(B) Dataset 2 (human gut microbiome)	
IBD (Gevers *et al.* 2014, Halfvarson *et al.* 2017)	993
HIV (Lozupone *et al.* 2013, Dinh *et al.* 2015, Noguera-Julian *et al.* 2016)	360
CRC (Baxter *et al.* 2016)	120
EDD (Singh *et al.* 2015)	222
Healthy control (Peterson *et al.* 2009, Lozupone *et al.* 2013, Gevers *et al.* 2014, Dinh *et al.* 2015, Noguera-Julian *et al.* 2016, Halfvarson *et al.* 2017, McDonald *et al.* 2018)	1418

For comparative analysis, we evaluated PM-CNN alongside conventional ML approaches, including KNN, DT, SVM, XGBoost, and RF that have been already widely used in microbiome studies (Cammarota *et al.* 2020, Greener *et al.* 2022, Asnicar *et al.* 2023) and DL approaches such as regular CNN and phylogeny-based MDeep. In addition, since MDeep was originally designed as a binary classifier, we adapted it for multi-status classification by modifying the number of neurons in its output layer while retaining the original model structure. We conducted cross-validation on the two test datasets. Each dataset was randomly split into a 70% training set and a 30% test set, and this process was repeated 10 times to ensure robustness of the results. Performance metrics for status classification were evaluated using various measures, including the Kappa coefficient, Recall, Accuracy, Precision, and F1 score (refer to the Section 5 for details).

### 3.2 Classification results of the human oral microbiome dataset

**Figure 4.**
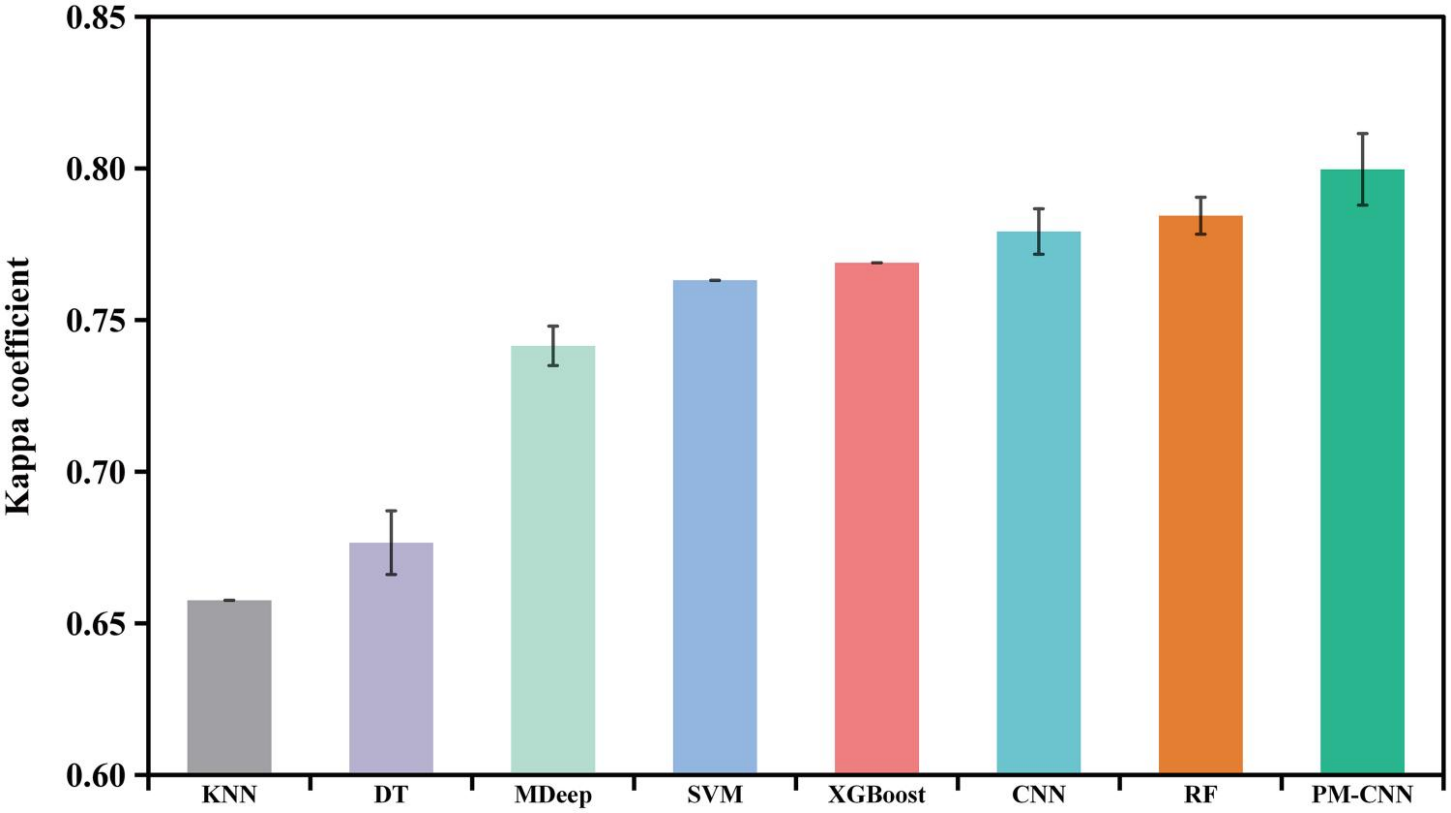
Kappa coefficients based on oral microbiomes of Dataset 1. Error bars represent the standard deviation of the 10 repeats.

Following microbiome profiling and essential curation, a total of 1554 OTUs were selected for model training and testing within Dataset 1. Subsequently, we constructed a phylogenetic tree encompassing all OTUs, from which we calculated the phylogenetically induced correlation structures. The comparative analysis of PM-CNN with the aforementioned methods was presented in Fig. 4 and Table 2 (original results are available in Supplementary Table S1). PM-CNN exhibited superior performance on both Kappa and F1 scores when compared to the single-branch convolution-based MDeep and CNN. Furthermore, among the traditional machine learning models, RF achieved the highest scores. Although PM-CNN had only a slight improvement in overall Kappa coefficient compared to RF, it exhibited a more balanced performance in the classification task of each individual status (e.g. lowest standard deviation and variance of F1 scores; see Supplementary Table S2 for details). This outcome underscored the effectiveness of PM-CNN in harnessing the phylogenetic relationships among microbes for classification tasks, filling the gap left by the traditional machine learning models, which lacked a priori knowledge of microbe-microbe relations.

**Table 2.** Results based on oral microbiomes of Dataset 1.

	Accuracy^a^	Recall^a^	Precision^a^	F1 score^a^
KNN	0.7883	0.7516	0.7858	0.7639
DT	0.7956	0.7995	0.7872	0.7926
MDeep	0.8379	0.8314	0.8330	0.8320
SVM	0.8532	0.8275		0.8425
XGBoost	0.8533	0.8586	0.8412	0.8457
CNN	0.8599	0.8651	0.8486	0.8536
RF	0.8632	0.8671	0.8525	0.8557
PM-CNN			0.8623	

a Mean values of 10 repeats.Bold values represent the maximum values.

### 3.3 Classification results of the human gut microbiome dataset

**Figure 5.**
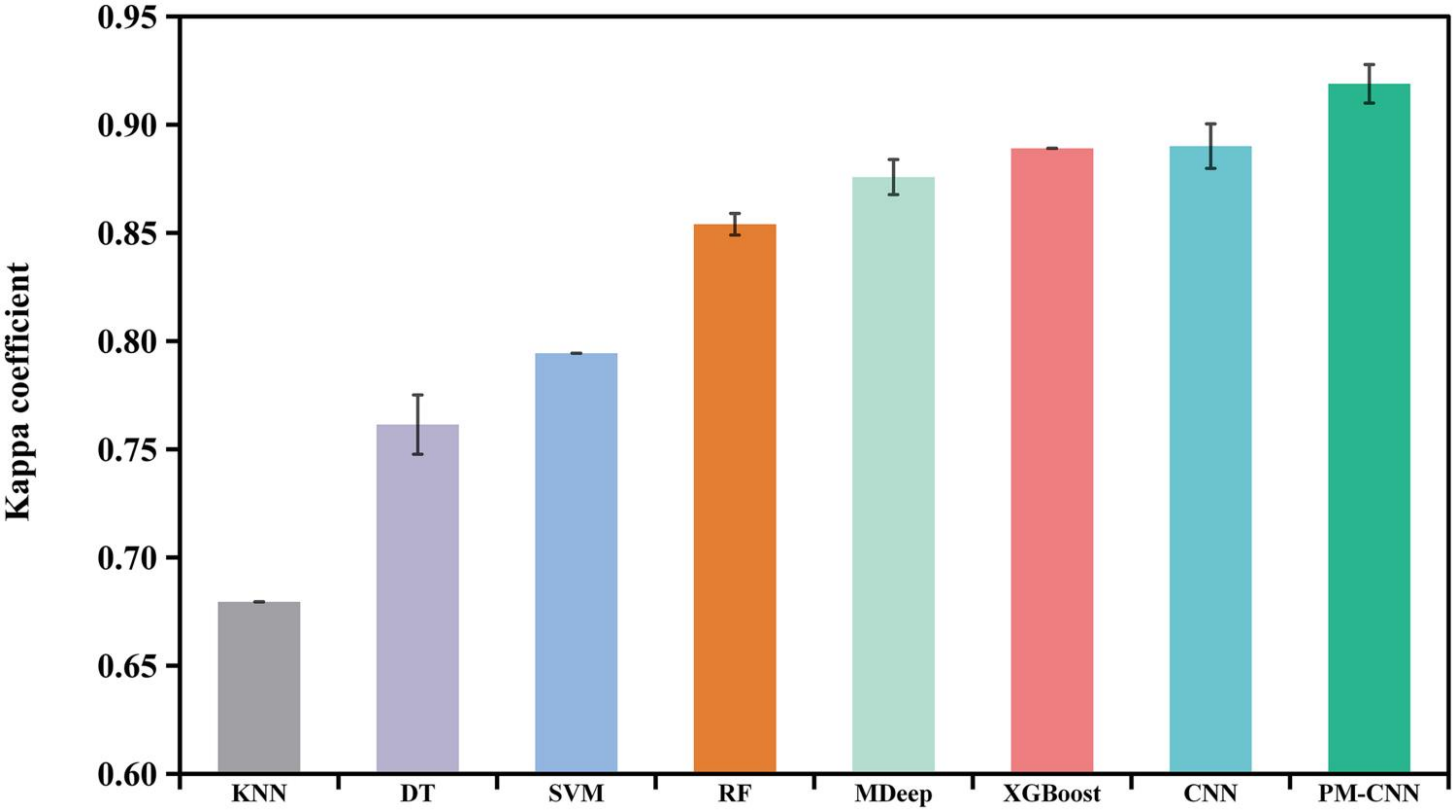
Kappa coefficients based on gut microbiomes of Dataset 2. Error bars represent the standard deviation of the 10 repeats.

To further validate the classification performance of PM-CNN, we employed Dataset 2, which consisted of 5597 OTUs extracted from 3113 human gut microbiomes. As demonstrated in the results presented in Fig. 5 and Table 3 (original results are available in Supplementary Table S3), PM-CNN consistently achieved the highest Kappa coefficient and outperformed other indices as well. In addition, PM-CNN also showed a stable accuracy in distinguishing different health status with lowest variance of F1 scores (see Supplementary Table S4 for details). Notably, with the increase in both the number of samples and status categories, the ranking of RF exhibited a significant decline across all models. Consequently, when we consolidated the outcomes from both datasets, it became evident that models based on neural networks consistently demonstrated superior performance compared to traditional machine learning methods.

**Table 3.** Results based on gut microbiomes of Dataset 2.

	Accuracy^a^	Recall^a^	Precision^a^	F1 score^a^
KNN	0.7837	0.7514	0.8019	0.7728
DT	0.8381	0.7940	0.8202	0.8018
SVM	0.7944	0.8332	0.8973	0.8585
RF	0.9002	0.9049	0.9218	0.9116
MDeep	0.9142	0.9425	0.9119	0.9255
XGBoost	0.9293	0.9366	0.9438	0.9295
CNN	0.9246	0.9348	0.9339	0.9334
PM-CNN				

a Mean values of 10 repeats.Bold values represent the maximum values.

## 4 Conclusion and discussion

In this study, we introduce PM-CNN (Phylogenetic Multi-path Convolutional Neural Network), a novel approach for microbiome-based multi-status classification and disease detection. Leveraging phylogenetic relationships among microbes, PM-CNN demonstrates remarkable performance across human oral and gut microbiomes, outperforming both traditional machine learning models and single-branch convolutional neural networks like MDeep. Our results underscore the significance of incorporating phylogeny into microbiome analysis, as PM-CNN effectively harnesses microbial evolutionary information for improved classification accuracy. This work not only contributes to the field of microbiome analysis but also highlights the importance of considering evolutionary relationships among microorganisms when tackling complex classification challenges. Future studies can explore PM-CNN's utility in a broader range of microbiome datasets and investigate its applicability in real-world clinical settings, paving the way for enhanced disease diagnosis and treatment strategies based on microbial data.

The efficacy of PM-CNN necessitates further validation across a broader spectrum of microbiomes and scrutiny through artificial microbiomes. However, the simulation of microbiomes remains a formidable challenge, hindered by the intrinsic complexity of microbiome data and the concealed intricacies of microbial–disease interactions and associations. It is crucial to acknowledge the absence of a one-size-fits-all methodology for all microbiome datasets, given the considerable diversity in microbial biomarker patterns across different statuses. Recent investigations reveal instances where diseases selectively impact a minute fraction of microbial members within a complex community, rendering their detection challenging on a holistic microbiome level. In addressing such scenarios, the recently proposed “local-alignment” beta-diversity dissimilarity (Zhang *et al.* 2023) emerges as a promising alternative to conventional machine learning or deep learning approaches, offering a nuanced solution tailored to the unique challenges posed by specific disease-microbiome interactions. This underscores the imperative need for a nuanced and context-aware approach in the application of computational methods to microbiome research.

## 5 Methods

### 5.1 Implementation of PM-CNN and other machine learning competitors

PM-CNN was developed by Python PyTorch package (Paszke *et al.* 2019). The source code, usage and resources were available at the Github repository at https://github.com/qdu-bioinfo/PM_CNN. MDeep and CNN were implemented by Python PyTorch package. XGBoost was implemented using the Python xgboost package (Chen *et al.* 2016) with default parameter settings. RF, SVM, DT, and KNN were implemented using the Python scikit-learn package (Pedregosa *et al.* 2012) with default parameter settings.

### 5.2 Microbiome profiling

The sequencing data of all microbiome samples were analyzed into OTUs (operational taxonomic units) using Parallel-Meta Suite software (Chen *et al.* 2022) and GreenGenes database (DeSantis *et al.* 2006). Relative abundances of OTUs were normalized and corrected by the 16S rRNA copy number. To reduce the sparsity of the input data, in each dataset, we dropped OTUs that had a 0-sequence count in more than 90% samples.

### 5.3 Phylogeny tree

The phylogeny can either be constructed from representative sequences (e.g. marker gene or amplicon), or provided by users from a NEWICK format file (e.g. for shotgun metagenome). In this work, the phylogeny tree was constructed by the representative sequences obtained from the GreenGenes database. Specifically, after completing the profiling step, representative sequences of OTUs were aligned using the Mafft software (Katoh *et al.* 2002). Then we used FastTree tool (Price *et al.* 2009) to construct the phylogenetic tree.

### 5.4 Cophenetic distance

**Figure 6.**
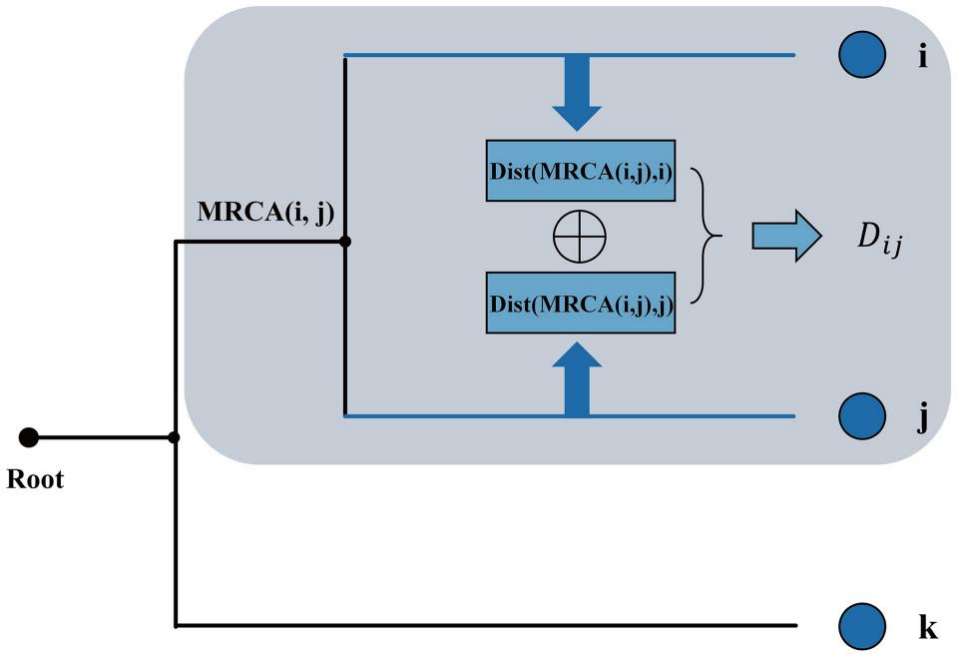
Cophenetic distance between two microbes in a phylogeny tree.

To calculate the normalized relations between microbes in a phylogeny tree, here we employed Cophenetic distance that measured the distance between microbes based on the heights of shared ancestral nodes. Specifically, this distance was computed as the sum of distances between each pair of tip nodes in the phylogenetic tree to their most recent common ancestor (MRCA; Fig. 6) as Equation (2):

(2) $$D_{ij}=\mathrm{Dist}\left(\mathrm{MRCA}\left(i,j\right),\;i\right)+\;\mathrm{Dist}\left(\mathrm{MRCA}\left(i,j\right),\;j\right).$$

Thus, considering evolutionary relationships in the phylogenetic tree, this computational approach enabled a quantitative assessment of dissimilarity between microbial groups or gene sequences.

### 5.5 Benchmark scores of multi-classification models

The Kappa coefficient (Cohen 1960) is an evaluation index based on the confusion matrix to measure the overall performance of multiple classification (Equation 3), which it is suitable for the imbalance of the number of samples in each category. The Kappa coefficient is the sum of the number of correctly classified samples of each class divided by the total number of samples. Assume that the number of real samples of each class is a_1_, a_2_,…, a_c_, and the number of samples of each class predicted is b_1_, b_2_,…, b_c_, and the total number of samples is *n*, $p_{e}=\frac{\left(a_{1}\times b_{1}+a_{2}\times b_{2}+\cdots +a_{c}\times b_{c}\right)}{n\times n}$.

(3) $$\mathrm{Kappa}=\frac{p_{o}-p_{e}}{1-p_{e}}.$$

(4) $$\mathrm{Recall}=\frac{\mathrm{TP}}{\mathrm{TP}+\mathrm{FN}}.$$

(5) $$\mathrm{Precision}=\frac{\mathrm{TP}}{\mathrm{TP}+\mathrm{FP}}.$$

(6) $$\mathrm{Accuracy}=\frac{\mathrm{TP}+\mathrm{TN}}{\mathrm{TP}+\mathrm{TN}+\mathrm{FP}+\mathrm{FN}}.$$

(7) $$F1\;\mathrm{score}=\frac{2\times \mathrm{Precision}\times \mathrm{Recall}}{\mathrm{Precision}+\mathrm{Recall}}.$$

We also calculate indices of binary classification to measure the performance of different models. Recall (Equation 4) is the proportion of correctly predicted TPs (Ture Positive) among all real positives (TP and FP; FP: False Positive). Precision (Equation 5) is the proportion of correctly predicted TPs among all predicted positives (TPs and FNs; FN: False Negative). Accuracy (Equation 6) is the proportion of correctly predicted samples (TP and TN; TN: Ture Negatives) among all samples (including TP, TN, FP and FN). F1 score integrates both Precision and Recall (Equation 7). In multiple classification tasks, these indices are calculated by the mean values of multiple categories.

## Supplementary Material

vbae013_Supplementary_Data

## Data Availability

The source code of PM-CNN and the datasets used in this work are available the Github repository at https://github.com/qdu-bioinfo/PM_CNN.
